# Remnant cholesterol, but not other cholesterol parameters, is associated with gestational diabetes mellitus in pregnant women: a prospective cohort study

**DOI:** 10.1186/s12967-023-04322-0

**Published:** 2023-08-07

**Authors:** Yajing Gao, Yanhua Hu, Lan Xiang

**Affiliations:** 1 Department of Anesthesiology, Affiliated Shenzhen Maternity and Child Healthcare Hospital, Southern Medical University, Shenzhen, 518028 China; 2 College of Information Science and Engineering, Liuzhou Institute of Technology, No. 99, Xinliu Avenue, Yufeng District, Liuzhou, 545616 Guangxi Zhuang Autonomous Region China; 3 School of Medical Technology and Nursing, Shenzhen Polytechnic, No.113, Tongfa Road 113, Nanshan District, Shenzhen, 518055 Guangdong China

**Keywords:** Gestational diabetes mellitus, Residual lipids, Predictors, Lipoprotein cholesterol, ROC

## Abstract

**Objective:**

No evidence has been found of a relationship between remnant cholesterol (RC) and the likelihood of gestational diabetes mellitus (GDM) in pregnant women. The aim of our study was to investigate the link between serum RC at 12–14 weeks of gestation and the risk of GDM.

**Methods:**

This was a secondary analysis with data from a prospective cohort study in Korea. A total of 590 single pregnant women attending two hospitals in Korea, up to 14 weeks gestation, from November 2014 to July 2016 were included in the study. The formula used to calculate RC in detail was RC (mg/dL) = TC (mg/dL)-HDL-c (mg/dL)-LDL-c (mg/dL). Logistic regression models were employed to examine the relationship between RC and GDM and explore the association between other lipoprotein cholesterol parameters and the risk of GDM. Furthermore, receiver operating characteristic (ROC) analysis was performed to assess the ability of RC to identify GDM. Additionally, sensitivity and subgroup analyses were conducted.

**Results:**

The mean age of participants was 32.06 ± 3.80 years. The median of RC was 34.66 mg/dL. 37 pregnant women (6.27%) were eventually diagnosed with GDM. Multivariate adjusted logistic regression analysis showed that RC was positively associated with the risk of GDM (OR = 1.458, 95% CI 1.221, 1.741). There was no significant association between other lipoprotein cholesterols (including TC, LDL-c, HDL-c) and the risk of GDM. The area under the ROC curve for RC as a predictor of GDM was 0.8038 (95% CI 0.7338–0.8738), and the optimal RC cut-off was 24.30 mg/dL. Our findings were demonstrated to be robust by performing a series of sensitivity analyses.

**Conclusion:**

Serum RC levels at 12–14 weeks of gestation are positively associated with GDM risk in pregnant women. RC in early pregnancy is an early warning indicator of GDM in pregnant women, especially those with normal HDL-c, LDL-c, and TC that are easily overlooked. There is a high risk of developing GDM in pregnant women whose RC is more than 24.30 mg/dL. This study may help optimize GDM prevention in pregnant women and facilitate communication between physicians, pregnant patients, and their families.

**Supplementary Information:**

The online version contains supplementary material available at 10.1186/s12967-023-04322-0.

## Introduction

Gestational diabetes mellitus (GDM) is characterized by elevated blood sugar levels first detected during pregnancy. GDM affects approximately 15% of pregnancies worldwide, accounting for approximately 18 million births annually [1–3]. GDM poses various risks to expectant mothers, including prenatal hypertension, pre-eclampsia, premature rupture of membranes, the birth of large-for-gestational-age babies, and an increased likelihood of cesarean section delivery [1, 4, 5]. Additionally, GDM heightens the chances of complications such as impaired carbohydrate metabolism, obesity, and cardiovascular disease. It also predisposes both the mother and the infant to the development of type 2 diabetes mellitus (T2DM) [6, 7]. The increased prevalence of GDM incurs significant economic costs, underscoring the need for heightened attention and awareness [8, 9]. Therefore, gaining an in-depth understanding of the risk factors associated with GDM is crucial.

GDM is influenced by various risk factors, including family history, age, dyslipidemia, obesity, and lack of physical activity [10, 11]. The relationship between dyslipidemia and GDM is controversial [12–14]. In one study, pregnant women with GDM exhibited higher serum lipid profiles compared to healthy pregnant women, including the ratios of low-density lipid cholesterol to high-density lipoprotein cholesterol (LDL-c/HDL-c ratio) and triglycerides to high-density lipoprotein cholesterol (TG/HDL-c ratio), as well as HDL-c levels [14]. However, other studies have reported no significant differences in serum levels of high-density lipoprotein cholesterol (HDL-c), total cholesterol (TC), triglycerides (TG), and low-density lipid cholesterol (LDL-c) between pregnant women with and without GDM [13, 15]. Recently, another marker, namely remnant cholesterol (RC), has been identified as being associated with an increased risk of cardiovascular diseases (CVD) and all-cause mortality [16, 17]. RC is characterized by lipoprotein cholesterol levels rich in TG, including intermediate-density lipoproteins and very low-density lipoprotein (VLDL) in the fasted state and celiac remnants in the non-fasted state [18]. Studies have shown that RC is significantly associated with the development of T2DM and that higher levels of RC not only increase the risk of microvascular complications but may also lead to macrovascular complications in diabetes [19–22]. In addition, recent studies have shown that RC predicts newly developed T2DM over traditional lipid parameters [19, 23, 24]. Based on these findings, we hypothesized that cholesterol levels, including RC, during early pregnancy might also be associated with GDM. Unfortunately, only a limited number of studies have explored the relationship between cholesterol parameters, especially RC, in early pregnancy and the risk of GDM. Therefore, a secondary analysis was conducted using published data to elucidate the association between cholesterol parameters and the risk of GDM.

## Methods

### Study design

A prospective cohort study design was used in this study. And data were obtained from a study, the "Fatty Liver in Pregnancy" registry (NCT02276144), conducted at the Government Seoul National University Boramae Medical Center and Incheon Seoul Women's Hospital [25].

### Data source

The dataset and information for this study were sourced from the publication titled "Nonalcoholic fatty liver disease is a risk factor for large-for-gestational-age birthweight" by Lee SM, Kim BJ, Koo JN, et al., published in PLoS ONE, volume 14, issue 8, with the article number e0221400 (2019). The publication is available under the Creative Commons Attribution License, allowing its unrestricted use, distribution, and reproduction, provided that proper credit is given to the author and source [25].

### Study population

Singleton pregnant women up to 14 weeks' gestation, who attended Seoul National University Boramae Medical Center and Incheon Seoul Women's Hospital from November 2014 to July 2016, were recruited by the original investigators as part of a prospective cohort study (ClinicalTrials.gov registry number: NCT02276144) [25]. The Institutional Review Board at Seoul National University Boramae Medical Center and the Public Institutional Review Board of the Korean Ministry of Health and Welfare both approved the original study's conduct. At the time of enrolment, every participant completed and signed an informed consent form [25]. Therefore, our secondary analysis did not require any further ethical clearance. The original study was also carried out in accordance with the Declaration of Helsinki; all procedures followed the necessary guidelines and rules, including the declaration section statement [25]. These same principles and guidelines apply to our secondary analysis as well.

**Fig. 1:**
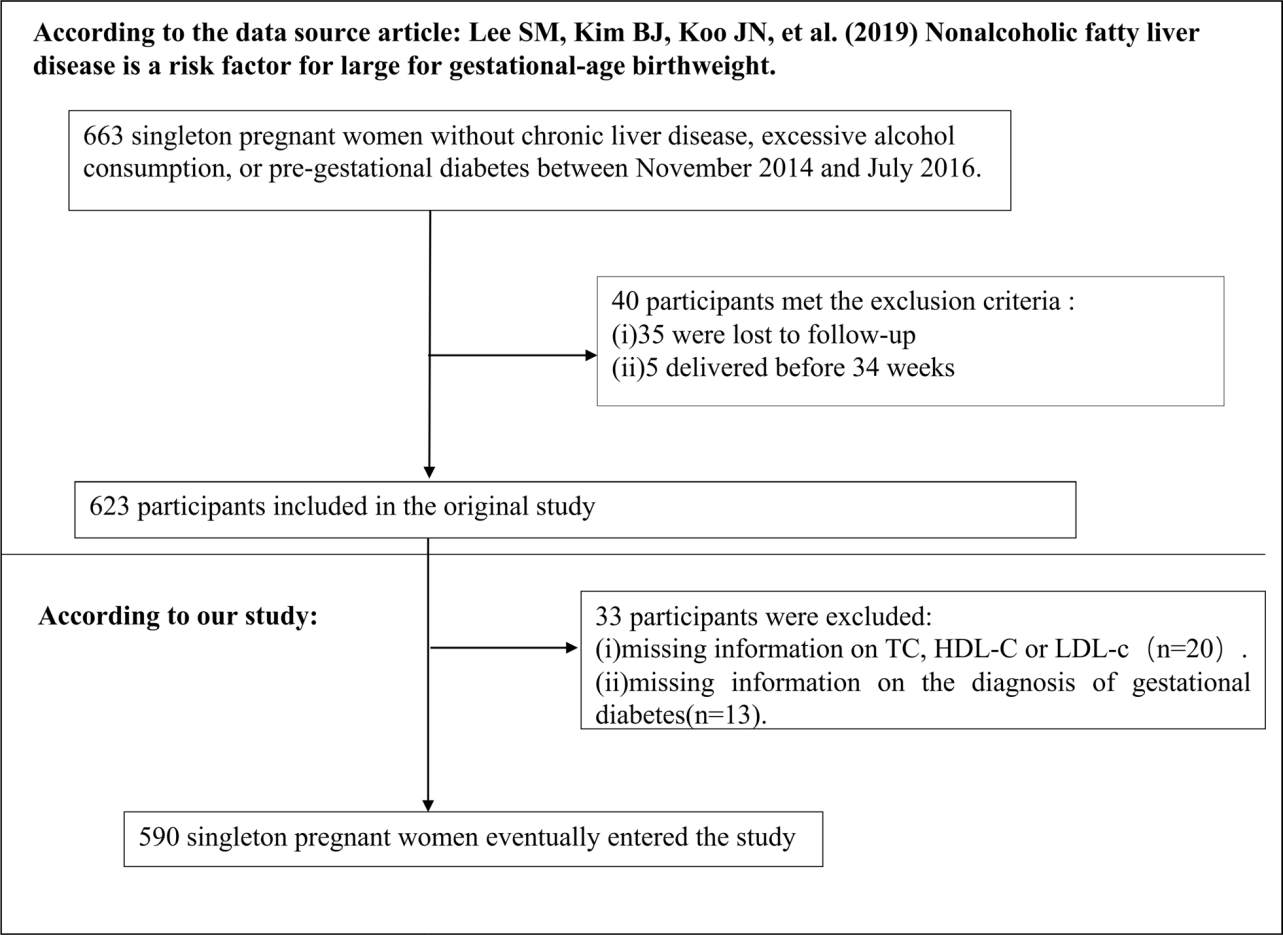
Flowchart of study participants

A total of 663 singleton pregnant women were initially recruited for the original study [25]. However, 40 participants were excluded from the analysis based on the following exclusion criteria: (1) evidence of pre-gestational diabetes, excessive alcohol consumption, or chronic liver disease; (2) loss to follow-up (n = 35); (3) preterm delivery before 34 weeks (n = 5). Consequently, the final number of participants in the original study was 623 [25]. For our present study, we further excluded 20 participants with missing information on TC, HDL-c, or LDL-c and 13 participants with unclear information on GDM. Ultimately, the study included 590 women with singleton pregnancies. The selection process for the participants is depicted in Fig. 1.

### Variables

#### Independent and outcome variables

RC was the target-independent variable. RC was recorded as a continuous variable in mg/dL. RC (mg/dL) = TC (mg/dL)-HDL-c (mg/dL)-LDL-c (mg/dL) is the formula used to calculate RC in detail [26, 27]. GDM (dichotomous: 0 = non-GDM, 1 = GDM) was the outcome variable.

### Covariates

The selection of covariates in our study was based on both the original study and our medical experience [25]. The following factors were taken into account as covariates: (1) continuous variable: age, pre-pregnancy body mass index (BMI), LDL-c, fasting plasma glucose (FPG), insulin, alanine, adiponectin, aminotransferase (ALT), high-density lipoprotein cholesterol to low-density lipid cholesterol ratio (HLR), TC, gamma-glutamyl transferase (GGT), aspartate aminotransferase (AST); (2) categorical variable: nulliparity, hepatic steatosis. Detailed information on the data collection and definition of variables can be found in the original study [25].

### Treatment of missing data

In the second analysis, the number of participants with missing data for pre-pregnancy BMI, FPG, insulin, AST, and ALT was 1 (0.17%), 1 (0.17%), 1 (0.17%), 2 (0.34%), and 2 (0.34%), respectively. In order to reduce the deviation caused by missing variables, which makes it impossible to accurately depict the statistical efficacy of the target sample during the modeling phase, multiple imputations are used for the missing data in this study [28, 29]. Age, pre-pregnancy BMI, hepatic steatosis, AST, GGT, ALT, FPG, adiponectin, and insulin were all taken into account in the imputation model (Iterations were 10, and the kind of regression was linear). Processes for missing data analysis employ the assumptions of missing-at-random (MAR) [28].

### Statistical analysis

The participants were stratified based on RC tertile. By ranking the RC values from smallest to largest and dividing them into three tertiles by frequency, each containing about one-third of the participants:: the lowest tertile (T1), the middle tertile (T2), and the highest tertile (T3), respectively [30–32]. For normally distributed continuous data, we give means and standard deviations; for skewed variables, we report medians; for categorical variables, we report percentages and frequencies. To examine statistical significance among RC groups, we used either a Kruskal–Wallis H test (for skewed distributions), One-way ANOVA (for normal distributions), or a χ2 test (for categorical variables).

### Logistic regression analysis

The link between RC and the risk of GDM was investigated using both univariate and multivariate logistic regression models. The multivariate logistic regression models were adjusted for various variables, including pre-pregnancy BMI, age, hepatic steatosis, AST, GGT, ALT, FPG, adiponectin, and insulin. Odds ratios (ORs) and 95% confidence intervals (CIs) were recorded in this study. Furthermore, the relationship between TG, TC, HDL-c, LDL-c, and HDL-c/LDL-c ratio (HLR) with GDM risk was explored using the same analytical approach. Additionally, variables demonstrating statistically significant differences in the univariate logistic regression analysis were further examined in the multivariate logistic regression models to assess their relationship with GDM risk.

### Subgroup analysis

A subgroup analysis was conducted using a stratified logistic regression model across various subgroups, including nulliparity, age, pre-pregnancy BMI, and HOMA-IR. Firstly, continuous variables, including pre-pregnancy BMI, age, and HOMA-IR, were converted into categorical variables based on clinical cut-off points (age: < 35, ≥ 35 years old; pre-pregnancy BMI: < 25, ≥ 25 kg/m^2^; HOMA-IR: ≤ 2, > 2) [33, 34]. Secondly, each stratification was adjusted for all other factors (age, pre-pregnancy BMI, hepatic steatosis, AST, GGT, ALT, FPG, adiponectin, insulin) except the specific stratification factor itself. Ultimately, the likelihood ratio test was used to determine whether interaction terms existed in models with and without interaction terms.

### Sensitivity analysis

Several sensitivity analyses were performed to assess the robustness of our findings. Firstly, RC was converted into a categorical variable based on tertiles. Due to the strong association between obesity, Non-alcoholic fatty liver disease (NAFLD), and GDM [25, 35], we excluded participants with pre-pregnancy BMI ≥ 25 kg/m^2^ or hepatic steatosis greater than or equal to grade 1 for further sensitivity analyses. Furthermore, an assessment was conducted to examine the potential presence of unobserved confounding between RC and the risk of GDM, utilizing the calculation of E-values. Previous research has indicated that the impact of unknown or unmeasured factors on the association between exposure and outcome is little when the E-value surpasses the relative risk of exposure and unmeasured confounders or when it falls below the association between unmeasured confounders and the outcome [36].

### Receiver operating characteristic curve analysis

Finally, a receiver operating characteristic (ROC) curve was generated to evaluate the predictive ability of TC, TG, LDL-c, and HDL-c in relation to the risk of GDM.

The analysis was carried out using Empower Stats (X&Y Solutions, Inc., Boston, MA, http://www.empowerstats.com) and the R statistical software tools (http://www.r-project.org, The R Foundation). Statistical significance was determined based on a two-sided approach, considering p-values below 0.05 as indicative of statistical significance.

## Results

### Characteristics of participants

**Fig. 2:**
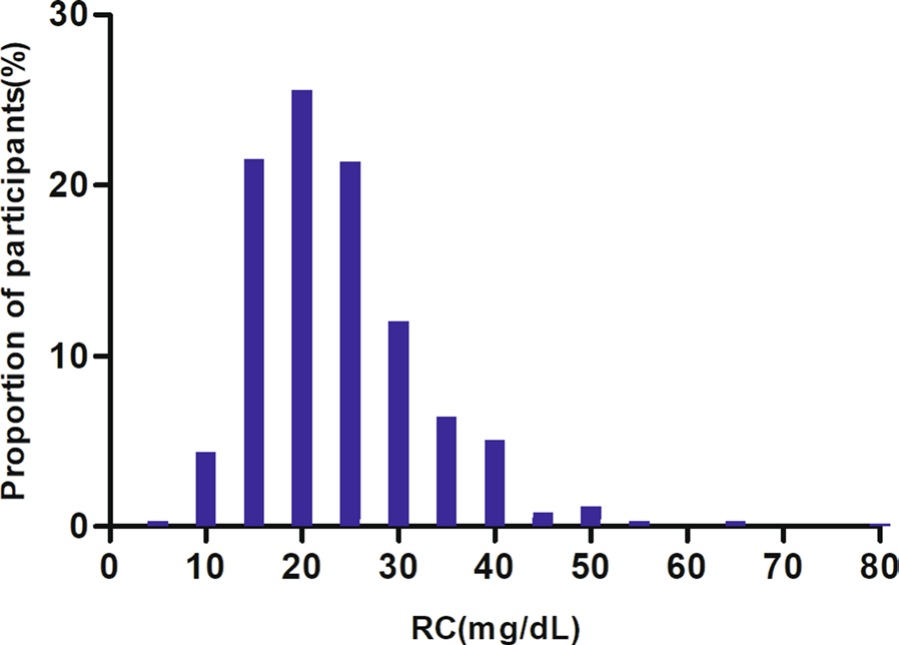
Distribution of RC. It presented a skewed distribution ranging from 2.6 mg/dL to 71.3 mg/dL, with a median (interquartile) of 34.66 (25.50,43.82) mg/dL

The study participants' demographic and clinical characteristics are presented in Table 1. The mean age was 32.06 ± 3.80 years old. The median of RC was 34.66 mg/dL. 37 (6.27%) pregnant women developed GDM. We divided participants into subgroups based on RC tertile (T1: < 18.50, T2:18.50–25.60, T3: ≥ 25.60 mg/dL). The highest tertile (T3: ≥ 25.60 mg/dL) showed significant increases in Pre-pregnancy BMI, TC, TG, LDL-c, GGT, FFA, HOMA-IR, and insulin in comparison with the lowest tertile (T1: < 18.50 mg/dL); however, HDL-c, HLR, and adipokines showed the opposite trend. Moreover, the highest tertile had a higher proportion of participants with nulliparity and steatosis grade 0. RC showed a skewed distribution with a median (interquartile) of 34.66 (25.50,43.82) mg/dL (Fig. 2). The presence or absence of GDM was used to split the participants into two groups. Additional file 1: Table S1 displayed the distribution of RC values in both the non-GDM and GDM groups. It is observed that the proportion of RC values < 25.6 mg/dL(T1) was higher in the non-GDM group. Conversely, the proportion of RC values ≥ 25.6 mg/dL(T2) was higher in the GDM group.

**Table 1:** The baseline characteristics of participants

RC	T1(< 18.50 mg/dL)	T2(18.50–25.60 mg/dL)	T3(> 25.60 mg/dL)	*P*-value
Participants	194	196	200	
Age(years)	31.61 ± 3.67	32.22 ± 3.60	32.35 ± 4.07	0.116
Pre-pregnancy BMI (kg/m^2^)	21.05 ± 2.68	21.80 ± 3.49	23.23 ± 3.87	< 0.001
GGT(IU/L)	11.00 (9.25–14.00)	11.00 (9.00–14.00)	13.00 (10.00–18.00)	< 0.001
TC (mg/dL)	161.24 ± 22.28	172.31 ± 25.41	184.68 ± 28.33	< 0.001
TG (mg/dL)	76.42 ± 12.71	109.54 ± 10.22	169.55 ± 44.69	< 0.001
HDL-c(mg/dL)	66.51 ± 11.91	65.96 ± 13.81	62.34 ± 14.42	0.004
LDL-c(mg/dL)	79.46 ± 19.13	84.44 ± 20.38	88.05 ± 24.59	< 0.001
HLR	0.90 ± 0.32	0.83 ± 0.30	0.77 ± 0.31	
RC (mg/dL)	15.27 ± 2.52	21.90 ± 2.04	34.29 ± 9.79	< 0.001
Adipokines(ng/mL)	6402.45 (3847.80–9978.28)	5482.90 (3196.80–8228.60)	3739.35 (2062.43–6010.28)	< 0.001
AST(IU/L)	16.00 (14.00–18.75)	16.00 (14.00–19.00)	17.00 (14.00–21.00)	0.085
ALT(IU/L)	11.00 (8.00–14.00)	11.00 (8.00–15.00)	11.50 (8.00–18.00)	0.068
FPG (mg/dL)	75.84 ± 8.95	77.14 ± 9.96	78.09 ± 10.17	0.058
Insulin(μIU/mL)	6.70 (4.32–10.07)	7.95 (5.30–10.93)	10.30 (7.30–15.07)	< 0.001
HOMA-IR	1.30 (0.80–1.90)	1.50 (0.90–2.20)	2.00 (1.30–2.82)	< 0.001
Nulliparity(n,%)				0.069
No	115 (59.28%)	98 (50.00%)	97 (48.50%)	
Yes	79 (40.72%)	98 (50.00%)	103 (51.50%)	
Hepatic steatosis(n,%)				< 0.001
Grade 0	167 (86.08%)	171 (87.24%)	141 (70.50%)	
Grade 1	27 (13.92%)	19 (9.69%)	39 (19.50%)	
Grade 2	0 (0.00%)	5 (2.55%)	13 (6.50%)	
Grade 3	0 (0.00%)	1 (0.51%)	7 (3.50%)	

Continuous variables were summarized as mean (SD) or medians (quartile interval); categorical variables were displayed as a percentage (%)

*RC* remnant cholesterol, *BMI* body mass index, *ALT* alanine aminotransferase, *AST* aspartate aminotransferase, *GGT* gamma-glutamyl transferase, *HDL-c* high-density lipoprotein cholesterol, *TC* total cholesterol, *TG* triglycerides, *LDL-c* low-density lipid cholesterol, *HLR* high-density lipoprotein cholesterol to low-density lipid cholesterol ratio, *FPG* fasting plasma glucose, *HOMA-IR* homeostasis model assessment-insulin resistance

### The incidence rate of gestational diabetes mellitus

In Table 2, it was presented that out of the total participants, 37 pregnant women developed GDM, resulting in an overall incidence of 6.27%. The incidence of GDM varied across the RC tertiles, with T1 having an incidence of 1.03%, T2 having an incidence of 3.06%, and T3 having the highest incidence of 14.50%. A significant trend was observed, indicating a higher prevalence of GDM among participants with the highest RC (T3) compared to those with the lowest RC (T1) (p for trend < 0.001) (Table 2).

**Table 2:** Incidence rate of gestational diabetes mellitus

RC	Participants(n)	GDM events (n)	Incidence of GDM (95% CI%)
Total	590	37	6.27 (4.31–8.23)
Tertile1	194	2	1.03 (0.40–2.50)
Tertile2	196	6	3.06(0.60–5.49)
Tertile3	200	29	14.50 (9.58–19.42)
*P* for trend			< 0.001

*RC* remnant cholesterol, *GDM* gestational diabetes mellitus, *CI* confidence interval

### The results of univariate analyses using a binary logistic regression model

The results of the univariate analyses revealed that there was no significant association between the risk of GDM and age (odds ratio [OR] = 1.036, 95% confidence interval [CI] 0.949–1.132), TC (OR = 1.011, 95% CI 0.999–1.023), LDL-c (OR = 1.001, 95% CI 0.985–1.016), HLR(OR = 0.733 95%CI0.240, 2.245), or AST (OR = 1.020, 95% CI 0.994–1.047) (all p > 0.05). However, there was a positive association between the risk of GDM and pre-pregnancy BMI (OR = 1.284, 95% CI 1.183–1.392), TG (OR = 1.019, 95% CI 1.012–1.025), RC (OR = 1.102, 95% CI 1.067–1.138), ALT (OR = 1.037, 95% CI 1.014–1.061), FPG (OR = 1.072, 95% CI: 1.039–1.106), insulin (OR = 1.123, 95% CI 1.073–1.175), and HOMA-IR (OR = 1.463, 95% CI 1.211–1.766) (all p < 0.05). Participants with steatosis grade 3 (OR = 17.362, 95% CI 3.814–79.042) and nulliparity (OR = 1.052, 95% CI 0.541–2.048) were also found to be more likely to experience GDM (all p < 0.05). Interestingly, adipokines (OR = 0.999, 95% CI 0.999–1.000) showed a negative relationship with the risk of developing GDM in pregnant women (Table 3).

**Table 3:** Factors influencing gestational diabetes based on a univariate logistic regression model

	Non-GDM (n = 553)	GDM (n = 37)	OR (95%CI)	*P-*valve
Age (years)	32.03 ± 3.81	32.54 ± 3.56	1.036 (0.949, 1.132)	0.43038
Nulliparity (n,%)				
No	291 (52.62%)	19 (51.35%)	Ref	
Yes	262 (47.38%)	18 (48.65%)	1.052 (0.541, 2.048)	0.881
Pre-pregnancy BMI (kg/m^2^)	21.78 ± 3.20	25.95 ± 5.17	1.284 (1.183, 1.392)	< 0.001
GGT (IU/L)	13.72 ± 8.38	18.81 ± 10.97	1.040 (1.014, 1.066)	0.002
TC (mg/dL)	172.32 ± 26.97	180.89 ± 29.61	1.011 (0.999, 1.023)	0.065
TG (mg/dL)	115.16 ± 41.67	176.22 ± 81.93	1.019 (1.012, 1.025)	< 0.001
HDL-c (mg/dL)	65.29 ± 13.25	59.29 ± 16.45	0.966 (0.941, 0.992)	0.009
LDL-c (mg/dL)	84.01 ± 21.29	84.29 ± 28.38	1.001 (0.985, 1.016)	0.939
HLR	0.84 ± 0.31	0.81 ± 0.40	0.733 (0.240, 2.245)	0.587
RC (mg/dL)	23.03 ± 8.34	37.31 ± 18.63	1.102 (1.067, 1.138)	< 0.001
Hepatic steatosis (n, %)				
Grade 0	463 (83.73%)	16 (43.24%)	Ref	
Grade 1	76 (13.74%)	9 (24.32%)	3.427 (1.462, 8.033)	0.005
Grade 2	9 (1.63%)	9 (24.32%)	28.937 (10.128, 82.677)	< 0.001
Grade 3	5 (0.90%)	3 (8.11%)	17.362 (3.814, 79.042)	< 0.001
Adipokines (ng/mL)	6289.62 ± 4296.63	2591.20 ± 2133.90	0.999 (0.999, 1.000)	< 0.001
AST (U/L)	17.71 ± 8.14	19.89 ± 7.34	1.020 (0.994, 1.047)	0.140
ALT (U/L)	11.00 (8.00–14.00)	15.00 (10.00–25.00)	1.037 (1.014, 1.061)	0.002
FPG (mg/dL)	76.54 ± 9.06	84.85 ± 15.10	1.072 (1.039, 1.106)	< 0.001
Insulin (μIU/mL)	8.10 (5.30–11.20)	15.30 (9.10–22.50)	1.123 (1.073, 1.175)	< 0.001
HOMA-IR	1.50 (1.00–2.20)	2.90 (1.60–4.60)	1.463 (1.211, 1.766)	< 0.001

Continuous variables were summarized as mean (SD) or medians (quartile interval); categorical variables were displayed as a percentage (%)

*RC* remnant cholesterol, *BMI* body mass index, *ALT* alanine aminotransferase, *AST* aspartate aminotransferase, *GGT* gamma-glutamyl transferase, *HDL-c* high-density lipoprotein cholesterol, *TC* total cholesterol, *TG* triglycerides, *LDL-c* low-density lipid cholesterol, *HLR* high-density lipoprotein cholesterol to low-density lipid cholesterol ratio, *FPG* fasting plasma glucose, *HOMA-IR* homeostasis model assessment-insulin resistance

*OR* odds ratio, *CI* confidence interval, *Ref* Reference

### Results of multivariate logistic regression analysis

Multivariate-adjusted logistic regression models were utilized to examine the association between RC and GDM in pregnant women (Table 4, Additional file 2: Figure S1). The findings revealed that, after accounting for confounding variables, there was a significant association between RC levels in early gestation and the risk of GDM in pregnant women. Specifically, for each 5 mg/dL increase in RC levels, there was a 45.8% increase in the risk of GDM (odds ratio [OR] = 1.458, 95% confidence interval [CI] 1.221–1.741). The confidence interval distribution further supports the independent association of RC with the risk of GDM.

**Table 4:** lipid parameters including TG, TC, LDL-c, HDL-c, HDL-c/LDL-c ratio, and RC in relation to gestational diabetes mellitus

Exposure	Non-GDM (n = 553)	GDM (n = 37)	Odds ratio (95% CI)	*P* value
TC (mg/dL)	172.32 ± 26.97	180.89 ± 29.61	+ 5 mg/dL: 1.035 (0.967, 1.107)	0.322
HDL-c(mg/dL)	65.29 ± 13.25	59.29 ± 16.45	+ 5 mg/dL: 0.880 (0.753, 1.028)	0.107
LDL-c(mg/dL)	84.01 ± 21.29	84.29 ± 28.38	+ 5 mg/dL: 0.990 (0.913, 1.073)	0.802
TG (mg/dL)	115.16 ± 41.67	176.22 ± 81.93	+ 5 mg/dL:1.068 (1.027, 1.111)	< 0.001
HDL-c/LDL-c ratio	0.84 ± 0.31	0.81 ± 0.40	1 unit: 0.985 (0.287, 3.377)	0.980
RC (mg/dL)	23.025 ± 8.337	37.305 ± 18.628	+ 5 mg/dL:1.458 (1.221, 1.741)	< 0.001
RC tertile				
T1 (< 18.50 mg/dL)	192 (34.72%)	2 (5.41%)	Ref	
T2 (18.50–25.60 mg/dL)	190 (34.36%)	6 (16.22%)	1.404 (0.254, 7.771)	0.698
T3 (≥ 25.60 mg/dL)	171 (30.92%)	29 (78.38%)	4.500 (0.973, 20.811)	0.054
*P* for trend				0.011

Values are mean ± SD or n (%) unless otherwise indicated. Odds ratios (OR) were estimated by multivariate-logistic regression models adjusted for age, pre-pregnancy BMI, hepatic steatosis, AST, GGT, ALT, FPG, adiponectin, and insulin

*CI* confidence interval, *Ref* Reference, *RC* remnant cholesterol

In addition, the association between GDM risk and other cholesterol parameters (LDL-c, TC, HDL-C, HLR) was examined (Table 4, Additional file 2: Figure S1). Through multivariate logistic regression analysis, it was found that the risk of GDM in pregnant women did not show a significant association with TC (per 5 mg/dL), HDL-c (per 5 mg/dL), LDL-c (per 5 mg/dL), and HLR after excluding confounding factors. The ORs, along with their corresponding 95% Cis and p-values, were as follows: TC (OR = 1.035, 95% CI 0.967–1.107, p = 0.322), HDL-c (OR = 0.880, 95% CI 0.757–1.028, p = 0.107), LDL-c (OR = 0.990, 95% CI 0.913–1.073, p = 0.802), and HLR (OR = 0.985, 95% CI 0.287–3.377, p = 0.980). In other words, there was no significant association observed between TC, HDL-c, LDL-c, and HLR with the risk of GDM in pregnant women.

Furthermore, the relationship between TG levels and GDM risk was further investigated using multivariate logistic regression models (Table 4, Additional file 2: Figure S1). The results revealed that after adjusting for confounding factors, there was a 6.8% increase in the risk of maternal GDM for every 5 mg/dL rise in TG levels during early pregnancy (OR = 1.068, 95% CI 1.027–1.111, p < 0.001). The relationship between other factors, including pre-pregnancy BMI, ALT, GGT, FPG, Insulin, HOMA-IR, and adipokines, and the risk of GDM was also investigated (Additional file 1: Table S2). Through multivariate logistic regression analysis, after excluding confounding factors, the odds ratios (ORs) along with their 95% confidence intervals (CIs) for the association between these factors and the risk of GDM were as follows: pre-pregnancy BMI (OR = 1.078, 95% CI 0.967–1.202), ALT (OR = 1.034, 95% CI 0.988–1.082), GGT (OR = 0.997, 95% CI 0.955–1.041), FPG (OR = 1.051, 95% CI 0.995–1.111), Insulin (OR = 1.145, 95% CI: 0.923–1.422), HOMA-IR (OR = 0.640, 95% CI 0.267–1.533), and adipokines (OR = 1.000, 95% CI 0.999–1.000). As observed from the confidence intervals, none of these factors were found to be significantly associated with the risk of GDM in pregnant women.

### Sensitivity analysis

A series of sensitivity analyses were conducted, including the introduction of the tertile of RC into the regression equation. The findings indicated that the overall trend in the effect sizes between the groups remained consistent with the results obtained when RC was treated as a continuous variable (p for trend = 0.011) (Table 4).

A sensitivity analysis was conducted on participants with pre-pregnancy BMI < 25 kg/m^2^. After adjusting for confounding variables, including pre-pregnancy BMI, age, hepatic steatosis, AST, GGT, ALT, FPG, adiponectin, and insulin, the results revealed a positive association between RC (per 5 mg/dL) and GDM in pregnant women (OR = 1.473, 95% CI 1.200–1.809, p < 0.001). Additionally, another sensitivity analysis was performed by excluding participants with hepatic steatosis greater than or equal to grade 1. After adjusting for confounding variables, including age, pre-pregnancy BMI, AST, GGT, ALT, FPG, adiponectin, and insulin, the findings indicated that RC (per 5 mg/dL) remained positively associated with the risk of GDM in pregnant women (OR = 1.511, 95% CI 1.211–1.885, p < 0.001) (Table 5). Furthermore, an E-value was calculated to assess the sensitivity of the link between RC and GDM risk in pregnant women to unmeasured confounders (Additional file 1: Table S3). The E-value of 2.28, which exceeded the relative risk of RC and unmeasured confounders (1.75) but was lower than the relative risk of unmeasured confounders with GDM (3.74), suggested that the influence of unknown or unmeasured factors on the association between RC and GDM risk is likely minimal. In addition, subgroup analyses were also done. In all analyzed predetermined or exploratory subgroups (Additional file 1: Table S4), there was no significant interaction between age, nulliparity, and HOMA-IR and RC (*P* for interaction > 0.05). Evidently, based on all sensitivity assessments, our conclusions are solid.

**Table 5:** Relationship between RC and GDM risk in different sensitivity analyses

	Exposure	Participants(n)	Non-GDM	GDM	Odds ratio (95% CI)	*P* value
Model I	RC (mg/dL)	492	22.51 ± 8.32	38.95 ± 22.48	+ 5 mg/dL: 1.473 (1.200, 1.809)	< 0.001
Model II	RC (mg/dL)	479	23.03 ± 8.34	37.31 ± 18.63	+ 5 mg/dL: 1.511 (1.211, 1.885)	< 0.001

Model I was a sensitivity analysis after excluding those with pre-pregnancy BMI ≥ 25 kg/m^2^. We adjusted for age, pre-pregnancy BMI, hepatic steatosis, AST, GGT, ALT, FPG, adiponectin, and insulin

Model II was a sensitivity analysis, including those with grade 0 hepatic steatosis. We adjusted for age, pre-pregnancy BMI, AST, GGT, ALT, FPG, adiponectin, and insulin

### The results of the ROC curve analysis

**Fig. 3:**
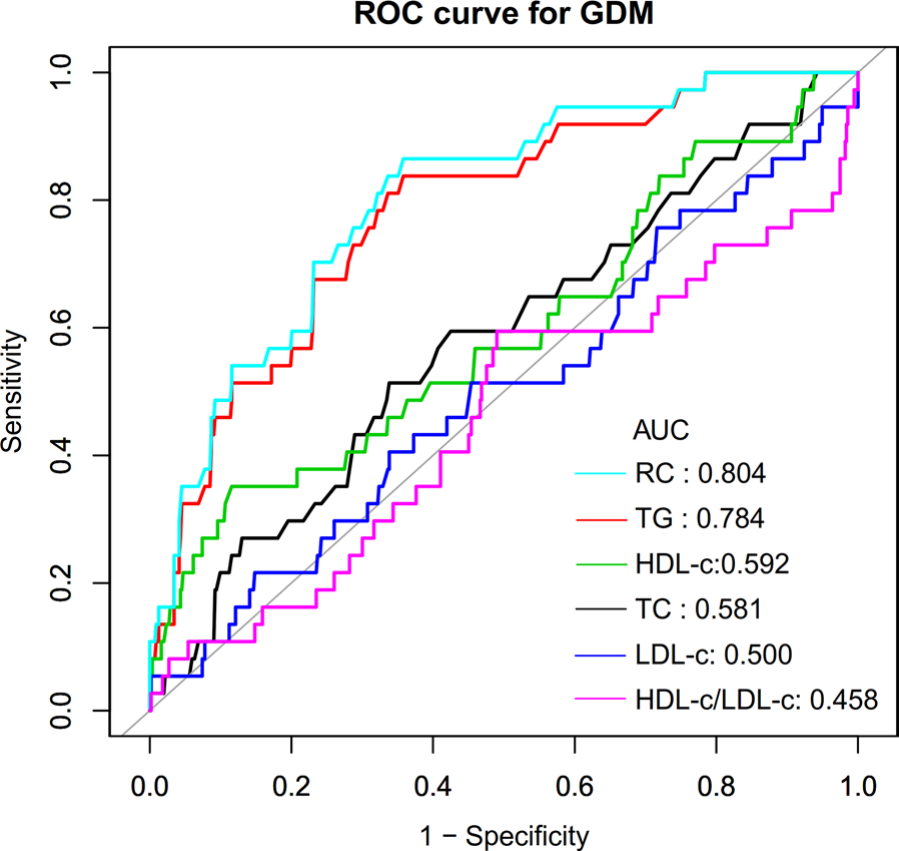
The results of ROC curve analysis for measuring the ability of RC, HDL-c, LDL-c, TG, HLR, and TC to predict the risk of GDM. Showed that RC was a better predictor of GDM in pregnant women than other lipid parameters. ROC curve analysis showed that the area under the curve for HLR, LDI-c, TC, HDL-c, TG, and RC were 0.4585, 0.5000, 0.5810, 0.5923, 0.7837, and 0.8038, respectively

We drew ROC curves to measure the ability of LDL-c, HDL-c, TG, TC, HLR, and RC to predict the risk of GDM in pregnant women (Fig. 3). The areas under the curve (AUC) of each variable were as follows: HLR:0.4585 < LDL-c: 0.5000 < TC: 0.5810 < HDL-c: 0.5923 < TG:0.7837 < RC:0.8038. The highest Youden index of HLR, LDL-c, TC, HDL-c, TG, HDL-c, LDL-c, and RC was 0.1045, 0.0679, 0.1753, 0.2357, 0.4798, and 0.5069, and the corresponding optimal cut-off value was 0.7633, 105.55, 181.50, 49.20, 121.50, 24.30, respectively. The Youden index and AUC of RC were the biggest, so the predictive ability of RC to GDM in pregnant women was better than that of other lipid parameters (Table 6).

**Table 6:** Area under the receiver operating characteristic curve (AUROC) for various lipid parameters for identifying GDM

Variables	AUC (95% CI)	Best threshold	Specificity	Sensitivity	Youden index
TC (mg/dL)	0.5810 (0.48226–0.6794)	181.5000	0.6618	0.5135	0.1753
TG (mg/dL)	0.7837 (0.7092–0.8582)	121.5000	0.6420	0.8378	0.4798
HDL-c(mg/dL)	0.5923 (0.4869–0.6977)	49.2000	0.8843	0.3514	0.2357
LDL-c(mg/dL)	0.5000 (0.3968–0.6032)	105.5500	0.8517	0.2162	0.0679
RC (mg/dL)	0.8038 (0.7338–0.8738)	24.3000	0.6420	0.8649	0.5069
HLR	0.4585 (0.3506- 0.5664)	0.7633	0.5099	0.5946	0.1045

*RC* remnant cholesterol, *HDL-c* high-density lipoprotein cholesterol, *TC* total cholesterol, *TG* triglycerides, *LDL-c* low-density lipid cholesterol, *HLR* high-density lipoprotein cholesterol to low-density lipid cholesterol ratio, *ROC* receiver operating characteristic curve, *CI* confidence interval

## Discussion

This prospective cohort study of 590 pregnant women examined the relationship between RC and GDM. The results found that RC was an independent risk factor for GDM in pregnant women but not for other cholesterol parameters, including HDL-c, LDL-c, and TC. The results of the sensitivity analysis further support a stable association between RC and the risk of GDM. We also demonstrated that RC can accurately predict GDM with an AUC of 0.8038 (0.7338–0.8738). RC was much superior to TG, HDL-c, TC, and LDL-c in predicting GDM in pregnant women. Therefore, RC may be an effective non-invasive method for predicting GDM.

Atherogenic dyslipidemia, characterized by elevated levels of TG, TG-rich lipoproteins, and decreased HDL-c levels, has been extensively studied in the context of cardiovascular disease [20, 37–39]. The association between cholesterol and cardiovascular disease remains a topic of debate. Some studies have concluded that cholesterol is not directly associated with cardiovascular disease but rather with inflammation. Some studies suggest that in the presence of atherosclerotic dyslipidemia, the overproduction of TG-rich lipoproteins and inefficient lipolytic processing contribute to increased RC formation. This cholesterol-enriched TG-rich lipoprotein is more prone to accumulate in the arterial wall, leading to atherosclerosis and cardiovascular disease [18, 40]. Conversely, other studies have concluded that cholesterol is not directly associated with cardiovascular disease but rather with inflammation [41, 42].However, there is growing evidence that RC is associated with T2DM and overall glucose metabolism [19, 24, 43, 44]. Considering that RC is one of the most prevalent patterns of lipid abnormalities in insulin-resistant states [24, 45, 46], we hypothesize that RC may affect pancreatic β cells and overall glucose metabolism in pregnant women. The current study investigated the association of RC with GDM and concluded that RC is an independent risk factor for the development of GDM in pregnant women, rather than other cholesterol parameters, including LDL-c, HDL-c, and TC. These findings are consistent with previous studies in the general population. Previous studies have shown that RC is positively associated with the risk of T2DM. In a longitudinal cohort study from Korea, the multivariate-adjusted analysis suggested that participants in the fourth quartile of RC had a higher risk of T2DM compared to those in the first quartile of RC (hazard ratios HR = 1.95;95% CI1.93–1.97). The HR for the second quartile of RC was 1.25 (95% CI1.24–1.27) and 1.51 (95% CI1.50–1.53) for the third quartile [19]. In addition, a single-center cohort study in China showed that higher RC levels were independently associated with an increased risk of new-onset diabetes (HR = 2.44, 95% CI 1.50–3.89) [24]. This is a very clinically relevant finding that RC is an independent risk factor for GDM and may be a potential target for the prevention and treatment of GDM. Clarifying their relationship informs the optimization of GDM prevention in pregnant women and facilitates communication with patients' families. It also provides valuable risk factors for future GDM prediction modeling.

Consistent with numerous previous studies, we employed the calculation of RC by subtracting HDL-c and LDL-c from TC, following the 2019 European Atherosclerosis Society guidelines for dyslipidemia management [21, 27, 47, 48]. Our study revealed a statistically significant difference in the association between RC and GDM when employing this simple calculation. However, none of the variables in the formula, including TC, LDL-c, and HDL-c, exhibited a significant difference in their association with GDM risk. Several possible reasons account for this discrepancy. Firstly, as suggested by Jepsen et al., calculated RC tends to slightly overestimate the directly measured values. Nonetheless, calculated RC remains closely linked to measured RC, and the advantage of the RC approximation lies in its wide availability and cost-effectiveness [17, 49]. Calculated RC can serve as a suitable alternative to measured RC for assessing the risk of various clinical outcomes [49]. However, it must be emphasized that the calculated RC remains essentially the cholesterol of all triglyceride-rich (TG) lipoproteins. There is growing evidence that abnormalities in TG, TG-rich lipoproteins, are associated with T2DM and overall glucose metabolism [43]. Studies have confirmed that elevated TG is strongly associated with an increased risk of GDM [50, 51]. Our study likewise confirmed a significant positive association between TG and the risk of GDM. Thus, the involvement of TG may be a reason for the significant positive association of RC with GDM. Secondly, the fact that RC remains cholesterol-specific in nature, it is more harmful to pancreatic β-cells because of its higher cholesterol content, quantity, and volume compared to LDL-c [52]. Additionally, RC may influence GDM through the inflammatory pathway, distinguishing it from conventional cholesterol. A study has provided evidence that elevated RC is causally associated with low-grade inflammation and ischemic heart disease (IHD), while elevated LDL-c is causally associated with IHD but not inflammation [53].

Previous studies have shown that in the general population, TG, HDL-c, and TC in the conventional lipid profile are good lipid parameters for predicting the risk of diabetes mellitus [24, 54, 55]. In the present study, HLR, LDL-c, TG, HDL-c, TC, and RC were calculated by ROC analysis in pregnant women at 12–14 weeks of gestation for their ability to predict GDM. The results showed that the area under the curve of RC was larger than other conventional lipid parameters. This suggests that RC may be a better indicator of glucose metabolism disorders in pregnant women and that measuring RC may help to predict pregnant women prone to GDM, especially those with normal HDL-c, LDL-c, and TC that are easily overlooked. When a pregnant woman's RC is greater than 24.3 mg/dL in early pregnancy, she has a high likelihood of developing GDM.

The underlying mechanism of the association between RC and GDM in pregnant women remains unclear. Insulin resistance may be the most critical factor. A study of residents in a rural community showed that fasting RC was strongly associated with IR [46]. In another study, postprandial RC was an independent predictor of IR, regardless of BMI, age, and other lipid profiles [56]. Additionally, RC may directly cause β-cell malfunction, which in turn causes insulin secretion suppression [57], and this may be one of the potential mechanisms of their relationship. Furthermore, the human placenta is an important site for the conversion of cholesterol to steroids, and the biosynthesis of placental steroids is essential for pregnancy maintenance and embryonic development [58]. In turn, a state of IR is induced in the second half of pregnancy by placental hormones such as placental-derived hormones, human placental lactogen, and human placental growth hormone [59, 60]. This may also be one of the mechanisms by which elevated RC in early gestation is associated with an increased risk of GDM.

There are several strengths to note in this investigation. (i) the study is the first in confirming the independent association between early plasma RC levels in pregnant women and GDM and making comparisons with concerns between other cholesterol parameters and GDM; (ii) our study provides an AUROC and optimal threshold for early prediction of GDM by RC; (iii) To ensure the robustness of the conclusions, a series of sensitivity analyses were performed, including the conversion of RC to a categorical variable, reanalysis of the association between RC and onset GDM after excluding participants with BMI ≥ 25 kg/m^2^ and hepatic steatosis greater than or equal to grade 1, subgroup analysis and calculation of E values to explore the possibility of unmeasured confounders.

The present study does have certain limitations. First, because the association between RC and GDM may vary by race, our findings should be further validated in different ethnic groups. Second, because this study was a secondary analysis, it was impossible to adjust for factors not in the original data set, such as waist circumference, family history of diabetes, and hypertension. In addition, the present study did not adjust for inflammatory markers such as platelet-activating factor, C-reactive protein, etc., and analyze their relationship with the risk of GDM. In the future, we can think about how to design our study, and we will obtain more precise data on variables, including some inflammatory mediators. The third drawback is that RC was collected only once at 12–14 weeks of gestation. Therefore, we do not know if the RC changes after 14 weeks. This is an important- issue that may require further study. Fourth, this observational study did not demonstrate a causal relationship between RC and GDM; rather, it only established an association. Finally, even if possible confounders were adjusted for, as with all observational research, there could still be unmeasured or uncontrolled confounders. We calculated E-value, nevertheless, and discovered that unmeasured or uncontrolled confounders had no impact on our results.

## Conclusion

In pregnant women, serum RC levels at 12–14 weeks of gestation are positively associated with GDM risk. Serum RC at 12–14 weeks of gestation predicted GDM better than other lipid markers, including TG, HDL-c, LDL-c, and TC. This study may help optimize GDM prevention in pregnant women and facilitate communication between physicians and pregnant women and their families.

### Supplementary Information

**Additional file 1: Table S1.** RC distribution in the non-GDM group and the GDM group. **Table S2.** Analysis of the relationship between selected factors and GDM based on multivariate logistic regression model. **Table S3.** The possibility for unobserved confounding between RC and the risk of GDM by calculating E values. **Table S4.** Effect size of RC on GDM in prespecified and exploratory subgroups.

**Additional file 2: Figure S1. **The forest plot for cox univariate analysis. showed that RC levels in early pregnancy were independently and positively associated with the risk of developing GDM in pregnant women after adjusting for confounders, whereas HLR, TC, HDL-c, and LDL-c were not significantly associated with the risk of GDM in pregnant women.

## Data Availability

The 'PLos one' database (which may be accessed at https://journals.plos.org/plosone/) allows users to save and download data.

## Abbreviations

RC: Remnant cholesterol
GDM: Gestational diabetes mellitus
ALT: Alanine aminotransferase
BMI: Body mass index
FPG: Fasting plasma glucose
AST: Aspartate aminotransferase
TG: Triglyceride
TG/HDL-c ratio: Triglyceride to high-density lipoprotein cholesterol ratio
HLR: High-density lipoprotein cholesterol to low-density lipid cholesterol ratio
HDL-c: High-density lipoprotein cholesterol
LDL-c: Low-density lipoproteins cholesterol
GGT: Gamma-glutamyl transferase
HDL-c: High-density lipoprotein cholesterol
TC: Total cholesterol
T2DM: Type 2 diabetes
GCT: Glucose challenge screening test
NAFLD: Non-alcoholic fatty liver disease
VLDL: Very low-density lipoprotein
ROC: Receiver operating characteristic
OR: Odds ratios
SD: Standard deviations
CI: Confidence interval

## Declarations

This research was authorized by the Institutional Review Board at Seoul National University Boramae Medical Center and the Public Institutional Review Board of the Korean Ministry of Health and Welfare and performed in line with the Declaration of Helsinki. Prior to enrolment, all individuals supplied informed permission in writing.
