# Supplementary material for: Remnant cholesterol, but not other cholesterol parameters, is associated with gestational diabetes mellitus in pregnant women: a prospective cohort study
Source: J Transl Med. 2023 Aug 7;21:531. doi: 10.1186/s12967-023-04322-0 (PMC10405385; doi:10.1186/s12967-023-04322-0)
Supplement: Supplementary file 1 — Additional file 1: Table S1. RC distribution in the non-GDM group and the GDM group. Table S2. Analysis of the relationship between selected factors and GDM based on multivariate logistic regression model. Table S3. The possibility for unobserved confounding between RC and the risk of GDM by calculating E values. Table S4. Effect size of RC on GDM in prespecified and exploratory subgroups. [file 12967_2023_4322_MOESM1_ESM.docx]

**Remnant cholesterol, but not other cholesterol parameters, is associated with gestational diabetes mellitus in pregnant women: a prospective cohort study.**

**Running title: RC and GDM**

Yajing Gao^1^, Yanhua Hu^2^*****, Lan Xiang^3^*****

1 Department of Anesthesiology, Shenzhen Maternity and Child Healthcare Hospital, Southern Medical University, Shenzhen 518048, China

2 College of Information Science and Engineering, Liuzhou Institute of Technology, Liuzhou 545616, Guangxi Zhuang Autonomous Region, China

3 School of Medical Technology and Nursing, Shenzhen Polytechnic, Shenzhen 518055, China.

***Corresponding author**

Yanhua Hu,

College of Information Science and Engineering,

Liuzhou Institute of Technology,

No. 99, Xinliu Avenue, Yufeng District

Liuzhou 545616,

Guangxi Zhuang Autonomous Region,

China

E-mail: 19418601@qq.com
***Corresponding author**

Lan Xiang,

School of Medical Technology and Nursing, Shenzhen Polytechnic,

No.113, Tongfa Road 113, Nanshan District,

Shenzhen 518055,

Guangdong Province

China

E-mail: Lanxiang_666@126.com

Table S1 RC distribution in the non-GDM group and the GDM group

|  | Non-GDM | GDM |
| --- | --- | --- |
| RC tertile |  |  |
| T1(<18.50mg/dL) | 178 (37.47%) | 2 (11.76%) |
| T2(18.50-25.60 mg/dL) | 168 (35.37%) | 3 (17.65%) |
| T3(T3: ≥25.60mg/dL) | 129 (27.16%) | 12 (70.59% |

Table S2 **Analysis of the relationship between selected factors and GDM based on multivariate logistic regression model**

| Exposure | Odds ratio (95% CI) p |
| --- | --- |
| Pre-pregnancy BMI (kg/m^2^) | 1.078 (0.967, 1.202) 0.17459 |
| ALT(U/L) | 1.034 (0.988, 1.082) 0.15351 |
| GGT(IU/L) | 0.997 (0.955, 1.041) 0.89291 |
| FPG (mg/dL) | 1.051 (0.995, 1.111) 0.07397 |
| Insulin(μIU/mL) | 1.145 (0.923, 1.422) 0.21872 |
| HOMA-IR | 0.640 (0.267, 1.533) 0.31708 |
| Adipokines(ng/mL) | 1.000 (0.999, 1.000) 0.0616 |

Note 1: Above model was adjusted for age, pre-pregnancy BMI, parity, hepatic steatosis, AST, GGT, ALT, TC, LDL-C, HOMA-IR, and adiponectin.

Note 2: The model was not adjusted for each of the independent variables themselves.

Table S3 The possibility for unobserved confounding between RC and the risk of GDM by calculating E values.

| Calculate E-value | relative risk of exposure-confounder | relative risk confounder-outcome |
| --- | --- | --- |
| 2.28 | 1.75 | 3.74 |

Table S4 Effect size of RC on GDM in prespecified and exploratory subgroups

| Characteristic | No. of patients | Effect size(95%CI) | P value | P for interaction |
| --- | --- | --- | --- | --- |
| Age (years) |  |  |  | 0.8273 |
| <35 | 453 | 1.087 (1.043, 1.133 | 0.0001 |  |
| ≥35 | 137 | 1.052 (0.986, 1.122) | 0.1239 |  |
| Pre-pregnancy BMI (kg/m^2^) |  |  |  | 0.5428 |
| <25 | 492 | 1.074 (1.031, 1.117) | 0.0005 |  |
| ≥25 | 97 | 1.094 (1.016, 1.179) | 0.0180 |  |
| nulliparity |  |  |  | 0.3029 |
| No | 310 | 1.100 (1.045, 1.158) | 0.0006 |  |
| Yes | 280 | 1.048 (0.994, 1.105) | 0.0169 |  |
| HOMA-IR |  |  |  | 0.5282 |
| ≤2 | 375 | 1.073 (1.031, 1.117) | 0.0006 |  |
| >2 | 215 | 1.095 (1.016, 1.181) | 0.0175 |  |

Note 1: Above model was adjusted for age, pre-pregnancy BMI, parity, hepatic steatosis, AST, GGT, ALT, TC, LDL-C, HOMA-IR, and adiponectin.

Note 2: The model is not adjusted for the stratification variable in each case.

Figure S1 The forest plot for cox univariate analysis.

Figure S1 showed that RC levels in early pregnancy were independently and positively associated with the risk of developing GDM in pregnant women after adjusting for confounders, whereas HLR, TC, HDL-c, and LDL-c were not significantly associated with the risk of GDM in pregnant women.
