# Supplementary figures and images for: Remnant cholesterol, but not other cholesterol parameters, is associated with gestational diabetes mellitus in pregnant women: a prospective cohort study
Source: J Transl Med. 2023 Aug 7;21:531. doi: 10.1186/s12967-023-04322-0 (PMC10405385; doi:10.1186/s12967-023-04322-0)

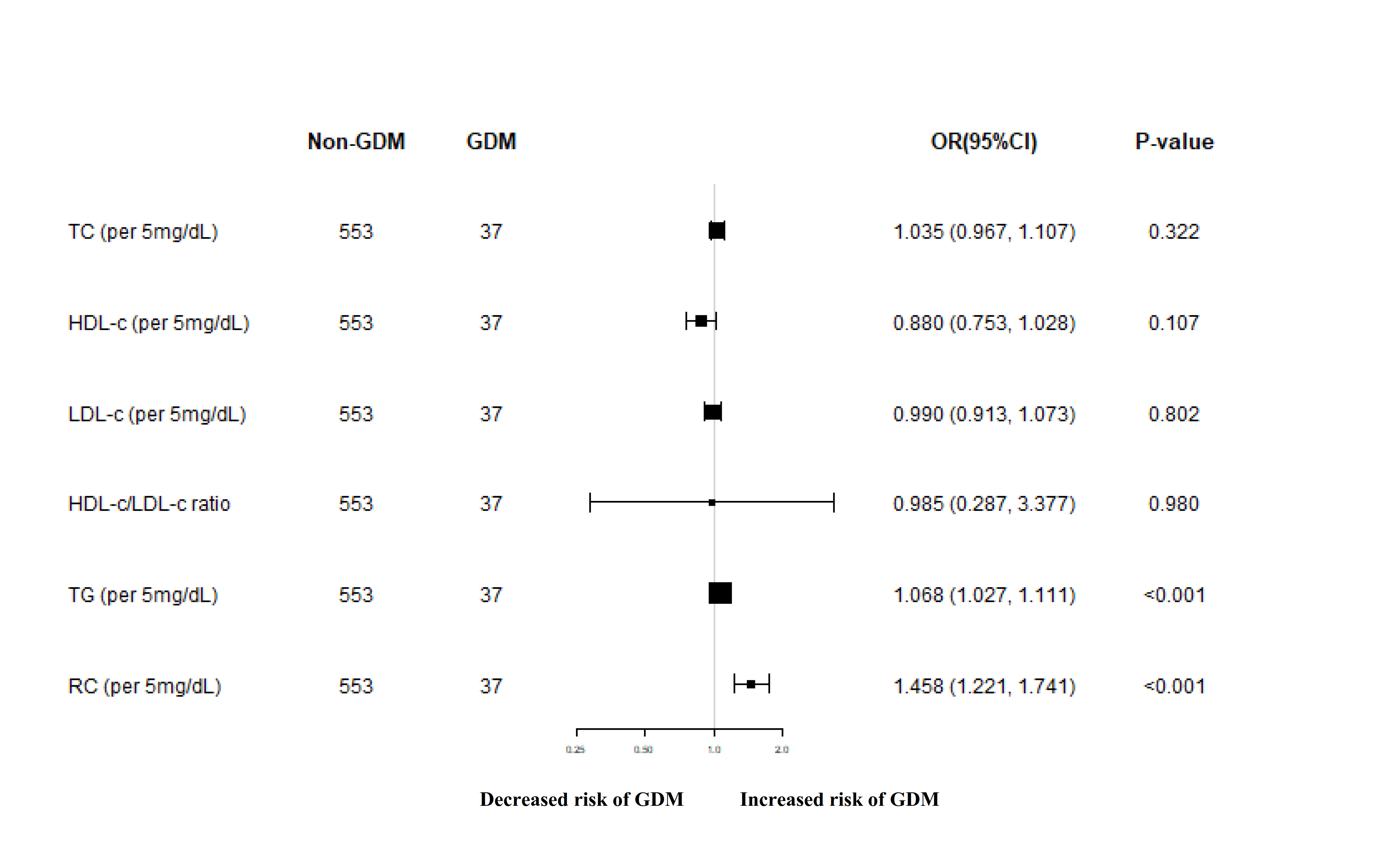

Supplement: Supplementary file 2 — Additional file 2: Figure S1. The forest plot for cox univariate analysis. showed that RC levels in early pregnancy were independently and positively associated with the risk of developing GDM in pregnant women after adjusting for confounders, whereas HLR, TC, HDL-c, and LDL-c were not significantly associated with the risk of GDM in pregnant women. [file 12967_2023_4322_MOESM2_ESM.tif]
